# A mechanism for cell non-autonomous inactivation of the tumor suppressor DAB2IP

**DOI:** 10.18632/oncoscience.441

**Published:** 2018-06-29

**Authors:** Arianna Bellazzo, Licio Collavin

**Affiliations:** National Laboratory CIB (LNCIB), AREA Science Park, 34149 Trieste, Italy; Department of Life Sciences, University of Trieste, 34127 Trieste, Italy

**Keywords:** microRNA, miR-149-3p, tumor microenvironment, vascular endothelial cells, NF-kB transcription factor

The dynamic crosstalk between cancer cells and tumor stroma is a major determinant of disease aggressiveness. Factors released by cancer cells (proteins, growth factors, cytokines, small RNAs) induce a stromal response involving fibroblast activation, intra-tumoral angiogenesis, and leukocyte infiltration; in turn, activated stromal cells remodel the extracellular matrix and release signals that can modify the behavior of cancer cells, e.g. promoting a mesenchymal-like phenotype [1, 2]. This reciprocal cell-to-cell communication profoundly affects tumor vascularization via crosstalk with endothelial cells [3], with implications for cancer growth and dissemination. In this context, signaling modulators that control specificity, amplitude, and duration of cell responses to external inputs can play a crucial role - often underestimated.

One such modulator is the tumor suppressor DAB2IP (Disabled 2 interacting protein), also known as AIP1 (Ask1 interacting protein). DAB2IP is a cytoplasmic Ras inhibitor (Ras-GAP) that also restrains NF-kB activation by inflammatory cytokines, and PI3K-AKT activation by various growth factors. More generally, DAB2IP acts as a cytoplasmic adaptor in signal transduction, negatively regulating multiple oncogenic pathways both in epithelial and in endothelial cells. Substantial evidence indicates that DAB2IP loss of function promotes epithelial to mesenchymal transition (EMT) and enhances invasiveness, proliferation, stemness, and chemoresistance in various cancer cell models [4, 5]. Accordingly, several mechanisms of DAB2IP inactivation have been described, ranging from promoter methylation to inhibitory interaction with mutant p53, to post-transcriptional downregulation by miRNAs [4, 5].

Performing a high-throughput functional screening, we identified a panel of microRNAs able to target the mRNA of DAB2IP. Among them, we characterized miRNA-149-3p as a potent antagonist of DAB2IP protein synthesis and DAB2IP oncosuppressive functions [6]. We observed that miR-149-3p expression in prostate cancer cell lines counteracts DAB2IP action, favoring an immunogenic and pro-angiogenic NF-kB-related gene expression program, and an invasive phenotype. We have successfully used LNAs (Locked Nucleic Acids) to mask the miR-149-3p seed sequences on DAB2IP mRNA, demonstrating that inhibition of miR-149-3p in malignant cells stabilizes DAB2IP protein levels and functions, and formally proving that the observed pro-oncogenic effects of miR-149-3p are mediated by its action on DAB2IP. *In vivo* xenograft experiments supported the *in vitro* results: inhibition of miR-149-3p counteracted prostate cancer cell dissemination in nude mice, and reduced cancer cell growth and angiogenic remodeling in zebrafish larvae [6].

Intriguingly, our functional screening identified both strands of miR-149 as positive hits: despite having different seed sequences, both miR-149-3p and miR-149-5p can target the DAB2IP mRNA. However, the consequences of their overexpression in tumor cells are different, since only miR-149-3p has a pro-invasive effect. We think this is due to the action of miR-149-5p on additional oncogenic cellular targets, counteracting tumor cell invasiveness [7]. Thus, cell behavior can be deeply affected by the relative concentrations of the two miR-149 strands. The expression of individual miRNA strands depends on molecular parameters controlling pri- and pre-miRNA processing, as well as co-factors modulating miRNA activity and stability; deregulation of such controls in human malignancies could contribute to the acquisition of oncogenic features by selectively potentiating miR-149-3p levels and functions.

In addition to inducing cell-autonomous effects, expression of miR-149-3p in cancer cells can possibly act also on the tumor microenvironment. Starting from evidence that miR-149-3p is secreted by cancer cells and can be detected in the plasma of melanoma patients [6, 7], we proved that secreted miR-149-3p induces DAB2IP downregulation in non-transformed cell lines and in primary endothelial cells, affecting their behavior and their response to secreted growth factors and cytokines. DAB2IP is abundantly expressed in endothelial cells, where its depletion increases proliferation, migration, tube formation capabilities, and vascular permeability [6, 8]. A recent study demonstrated that conditional DAB2IP knockout in vascular endothelial cells facilitates formation of a pre-metastatic niche, increasing tumor growth and dissemination in mouse models of melanoma and breast cancer [8]. These results imply that DAB2IP may have a relevant tumor-suppressive role in controlling the behavior of stromal cells; at the same time, they raise the question of whether DAB2IP is actually inactivated in tumor-associated vascular endothelial cells - and how.

We observed DAB2IP downregulation in endothelial cells treated with medium conditioned by prostate cancer cells. This is a possible mechanism by which cancer cells can inactivate DAB2IP in stromal cells, to establish a supporting microenvironment that could facilitate tumor growth and dissemination - exactly as modeled in mice by conditional knockout [6]. We found that miR-149-3p partially mediates this phenotype in prostate cancer cell lines, but additional factors are likely to be involved, including other DAB2IP-targeting miRNAs. Perhaps a variable cocktail of secreted miRNAs could modulate DAB2IP expression and functions in stromal cells of different tumor types. Also, the action of secreted miRNAs could integrate and complement the action of growth-factors and cytokines in the tumor microenvironment.

Further studies will be required to define mechanisms and impact of cell non-autonomous DAB2IP inhibition in endothelial cells, and possibly in other stromal cells. Results of these studies may provide additional insights on the reciprocal regulation between tumor and stroma in cancer progression, and may reveal novel targets for therapy.
